# Differential Expression of Human and Bacterial Proteins Reveals Microbiome–Host Crosstalk in Metabolic Disorders

**DOI:** 10.1002/prca.70054

**Published:** 2026-08-19

**Authors:** Carlos Vinicius Ferreira da Silva, Carlos José Ferreira da Silva, Fernanda da Silva Marinho, Youssef Bacila Sade, Sandra Mara Naressi Scapin, Fabiano L. Thompson, Cristiane Thompson, Eidy de Oliveira Santos

**Affiliations:** ^1^ Universidade Federal do Rio de Janeiro (UFRJ) Rio de Janeiro Rio de Janeiro Brazil; ^2^ Universidade Do Estado Do Rio de Janeiro (UERJ) Rio de Janeiro Rio de Janeiro Brazil; ^3^ Afya‐Universidade Do Grande Rio (Afya ‐Unigranrio) Duque de Caxias Rio de Janeiro Brazil; ^4^ Instituto Nacional de Metrologia Qualidade e Tecnologia (INMETRO) Duque de Caxias Rio de Janeiro Brazil

**Keywords:** host–microbiota, interactions, metabolic disorders, obesity and diabetes, salivary proteomics

## Abstract

**Purpose:**

The growing prevalence of obesity, MetS, and T2DM highlights the need to better understand host–microbiota interactions. While gut microbiota has been widely investigated, interactions between the oral microbiota and human salivary proteins remain largely unexplored.

**Experimental Design:**

This study investigated the integrated human salivary proteome and bacterial secreted metaproteome in Brazilian individuals spanning different metabolic states: normal weight/control, overweight, obesity, MetS, and T2DM. Saliva samples were analyzed using mass spectrometry‐based proteomics to identify differential protein profiles.

**Results:**

Key findings revealed significant downregulation of human proteins MYSM1 (eta2 = 0.710, 95% CI: [0.642, 0.825], qadj< 0.001) and GAD65 (*η* ^2^ = 0.478, 95% CI: [0.368, 0.681], qadj < 0.001) in obese, MetS, and T2DM groups, correlating negatively with BMI, waist circumference, and HOMA‐IR, suggesting impaired anti‐inflammatory and endocrine functions that exacerbate metabolic dysregulation. Conversely, carbonic anhydrase VI (CA6) was markedly upregulated (*η* ^2^ = 0.374, 95% CI: [0.274, 0.570], qadj < 0.001), showing positive correlations with systolic blood pressure and glucose levels, indicative of an acidic, inflammatory oral microenvironment linked to chronic low‐grade inflammation. In the bacterial secreted metaproteome, TrxC‐2 (*η* ^2^ = 0.557, 95% CI: [0.501, 0.727], qadj < 0.001), UMPK (*η* ^2^ = 0.629, 95% CI: [0.571, 0.781], qadj < 0.001), and RsmH (*η* ^2^ = 0.772, 95% CI: [0.718, 0.862], qadj 0.001) were significantly elevated in obesity, MetS, and T2DM, positively associated with anthropometric and insulin resistance markers, reflecting microbial adaptations to oxidative stress and enhanced virulence through biofilm formation and RNA biosynthesis. Interactome analysis demonstrated strong negative correlations between bacterial proteins and human proteins (MYSM1, GAD65), alongside positive correlations with CA6, unveiling a vicious cycle, where oral dysbiosis amplifies host inflammation and metabolic dysfunction, while altered human proteins perpetuate microbial imbalance.

**Conclusions and Clinical Relevance:**

These findings underscore the pivotal role of host–microbiota interactions in the oral cavity during the pathogenesis of obesity, MetS, and T2DM, highlighting the importance of further elucidating the molecular and functional mechanisms underlying microbiota–host crosstalk in metabolic diseases.

## 1 Introduction

Obesity is a chronic disorder, classified as a noncommunicable disease (NCD) by the WHO, characterized by an abnormal accumulation of adipose tissue, resulting in a series of responses mediated by the secretions of adipokines from this endocrine organ [1]. Generally, it presents as a multifactorial disease, influenced by a complex interplay of genetic, environmental, and behavioral factors [2]. A hallmark of obesity is its association with chronic low‐grade inflammation, which plays a pivotal role in the development and progression of numerous comorbidities, including cardiovascular diseases, metabolic syndrome (MetS), type 2 diabetes mellitus (T2DM), and dysbiosis [3, 4, 5].

**Clinical relevance**

This study demonstrates that saliva, a noninvasive and easily accessible biological fluid, can provide important molecular insights into metabolic diseases such as obesity, metabolic syndrome, and type 2 diabetes.

The identification of MYSM1 and GAD65 as negatively associated with BMI and metabolic impairment suggests their potential as indicators of compromised anti‐inflammatory regulation. In contrast, the upregulation of carbonic anhydrase VI (CA6), is positively associated with glucose levels and blood pressure. These findings support the idea that oral molecular profiles may reflect systemic metabolic status and could be explored in future screening approaches.

Furthermore, the increased abundance of bacterial proteins related to oxidative stress and virulence (TrxC‐2, UMPK, and RsmH) suggests that metabolic disorders may drive adaptive changes in the oral microbiota that contribute to inflammation. The observed host–microbiota interactions reinforce the clinical importance of oral health in metabolic diseases.

Recent global estimates indicate that over 1 billion individuals are living with obesity, including 880 million adults and 159 million children [2]. In Brazil, the prevalence of overweight and obesity reached 37% and 31%, respectively, among adults in 2024 [6]. This escalating trend poses a significant burden on healthcare systems worldwide, particularly in low and middle‐income countries such as Brazil, where the rise in obesity is closely linked to the increasing incidence of MetS, T2DM, and oral dysbiosis [7]. Metabolic Syndrome is a cluster of interrelated risk factors that elevate the likelihood of cardiovascular diseases, type 2 diabetes mellitus and stroke [8]. According to the National Cholesterol Education Program Adult Treatment Panel III (NCEP ATP III) criteria [9], MetS is defined by the presence of at least three of the following components: hypertriglyceridemia, reduced high density lipoprotein (HDL) cholesterol, elevated waist circumference, hypertension, and hyperglycemia. Insulin resistance or T2DM may also be included as diagnostic criteria [10, 11]. Globally, the prevalence of MetS was estimated at 12.5% in 2021 [12]. In Brazil, a study by De Siqueira Valadares [13] reported a concerning prevalence of 31% for MetS over the past decade (2011–2021), based on the NCEP ATP III criteria.

Obesity is widely recognized as a metabolic disorder characterized by impaired lipid, protein, and carbohydrate metabolism, resulting from insufficient insulin secretion by pancreatic β‐cells, insulin resistance, or a combination of both [14]. Insulin resistance is manifested by reduced responsiveness of insulin sensitive organs and tissues, resulting in decreased sensitivity to the hormone. In this context, the relationship between inflammatory conditions and insulin resistance has been extensively studied [15]. According to the International Diabetes Federation (IDF) Atlas [16], approximately 537 million adults were living with T2DM worldwide in 2021, with 75% residing in low‐ and middle‐income countries. The same year witnessed 6.7 million deaths attributable to diabetes (IDF, 2021). In Brazil, 15.7 million adults were diagnosed with T2DM in 2021, positioning the country as the sixth highest in the global ranking of diabetes prevalence.

Emerging evidence suggests that alterations in microbial composition are associated with various autoimmune and inflammatory conditions, including metabolic diseases. Among these, obesity, T2DM, and MetS are the most prevalent disorders characterized by metabolic dysregulation and concomitant oral microbiota dysbiosis [17, 18]. In this mutualistic host microorganism relationship, microbiomes arise as environments shaped by complex interactions between the genetic potential of hundreds of trillions of microorganisms and host tissues. Metaproteomics secreted has emerged as a powerful tool to explore not only microbial diversity and abundance but also the intricate interactions between the microbiota and the host proteome, including signal transduction pathways and metabolic networks [19].

In recent years, mass spectrometry‐based proteomics has emerged as a cornerstone approach for the differential analysis of various complex diseases, enabling the precise quantification of global protein expression shifts and the discovery of tissue specific molecular signatures in conditions ranging from oncology to chronic inflammatory disorders [20]. When applied to proximal biological fluids, this high throughput technology allows for non‐invasive dynamic profiling of both host responses and microenvironmental variations. In this context, recent studies have demonstrated significant alterations in the salivary proteome of individuals with these metabolic conditions, including the dysregulation of human proteins, such as cobalamin transporter, profilin‐1, alpha‐2‐macroglobulin, and ceruloplasmin, as well as bacterial proteins like amylase binding protein, lipoprotein TmpC, Efem/EfeO family lipoprotein AbpA, and Copper containing nitrite reductase [21, 22]. However, despite these isolated findings, the broader host–microbe signaling networks underlying these metabolic disorders within the salivary fluid remain largely underexplored [22, 23]. Therefore, a comprehensive, concurrent characterization of human and bacterial salivary proteomes by mass spectrometry can provide abundant, high‐dimensional data, deepening the understanding of how systemic metabolic modifications resulting from these progressive diseases can alter the functional dynamics between the host and the resident microbiota.

An integrated analysis of human and bacterial proteins in the saliva of healthy individuals and those with obesity, MetS, and 2DM could provide valuable insights into the complex mechanisms of host–microbiota interactions and their role in systemic metabolic progression. Therefore, this cross‐sectional study aimed to map specific structural alterations within the human and bacterial oral secreted metaproteome associated with increased BMI, obesity, and its secondary complications. By characterizing the interactome between the human salivary proteome and the oral bacterial secreted metaproteome, we sought to elucidate the functional crosstalk between the microbiome and the host under the pressure of metabolic stress. This primary goal was successfully achieved, leading to the identification of tightly coordinated, multi‐group host–microbial networks. Specifically, our findings revealed distinct patterns of activated and deactivated molecular pathways, characterized by the suppression of key host anti‐inflammatory proteins alongside the upregulation of bacterial stress response and virulence mechanisms, which unveil how oral dysbiosis and chronic low‐grade inflammation coevolve throughout the progressive stages of metabolic disorders.

## 2 Materials and Methods

### 2.1 Experimental Design

The study population comprised Brazilian adults aged 18 years or older, recruited from the state of Rio de Janeiro. Data collection was conducted in a clinical laboratory at the Afya‐Unigranrio University, located in Duque de Caxias, Rio de Janeiro, where saliva samples, anthropometric measurements, and biochemical tests were obtained in the morning following a 12‐h fasting period.

**FIGURE 1:**
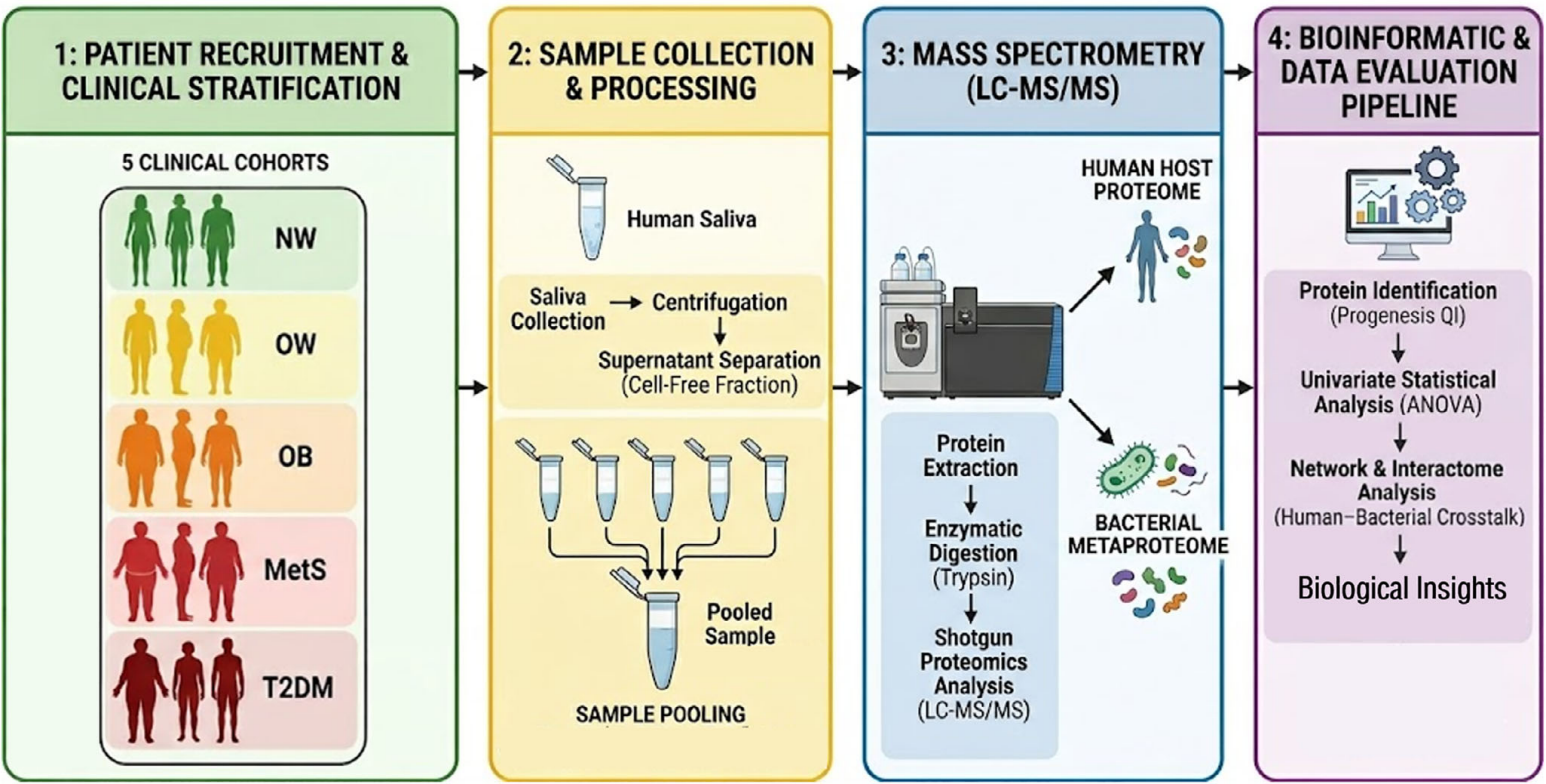
Comprehensive experimental and computational metaproteomic workflow. The discovery pipeline is structured into four sequential phases: (1) Patient recruitment & clinical stratification; (2) Sample collection & processing, consisting of human saliva collection, centrifugation for cell free fraction isolation, and subsequent group specific sample pooling; (3) Mass spectrometry, involving protein extraction, tryptic enzymatic digestion, and high‐resolution shotgun bottom up proteomics analysis to simultaneously capture the human host proteome and the oral bacterial metaproteome architectures; and (4) Bioinformatic & data evaluation Pipeline, where automated protein identification is performed via progenesis QI, followed by downstream univariate multi group statistical analysis (ANOVA), topological network interactome assessment to decode cross kingdom molecular crosstalk, and the extraction of core biological insights driving systemic metabolic progression.

Healthy individuals were stratified into three groups based on body mass index (BMI): normal weight/control (NW; *n* = 29; BMI ≥ 18.5 kg/m^2^ and ≤ 24.9 kg/m^2^), over‐weight (OW; *n* = 25; BMI ≥ 25 kg/m^2^ and ≤ 29.9 kg/m^2^), and obese (OB; *n* = 15; BMI ≥ 30 kg/m^2^). Additionally, the study included individuals diagnosed with metabolic syndrome (MetS; *n* = 23) and type 2 diabetes mellitus (T2DM; *n* = 11). Participants in the T2DM group had been previously diagnosed with the condition; thus, this study did not perform any diagnostic assessments for T2DM (Figure 1). The study protocol was approved by the Ethics in Human Research Committee of Unigranrio, RJ, Brazil (approval number 3,402,791). To be eligible for study inclusion, participants were stratified into distinct clinical cohorts based on their BMI thresholds and diagnostic criteria, as follows: normal weight (≥ 18.5 to 24.9 kg/m2), overweight (≥ 25.0 to 29.9 kg/m2), and obese (≥ 30.0 kg/m2). For the remaining experimental cohorts, eligibility required a formal clinical diagnosis of T2DM for the diabetes group, or meeting the established diagnostic criteria characterizing MetS. The exclusion criteria for the first study group were: presence of hepatopathy, nephropathy, endocrinopathies, neoplasms, cardiovascular diseases, autoimmune, hematological, psychiatric, inflammatory bowel diseases, pregnancy, lactation, and smoking, use of drugs that interfere with body weight, carbohydrate, lipid metabolism, and antihypertensives. Use of chemoprophylaxis for dental care, use of antibiotics in the last 6 months. Adult volunteers of both genders, aged 18 years or older with no upper age limit, were eligible for study inclusion. All participants provided written informed consent prior to enrollment, and the study was conducted in compliance with the ethical principles outlined in the 1964 Declaration of Helsinki and its subsequent amendments.

### 2.2 Clinical Evaluation

Clinical assessments were carried out as previously described. The evaluation encompassed anthropometric measurements, including body mass index (BMI), height, weight, waist circumference, hip circumference, and waist‐to‐hip ratio (WHR), as well as blood pressure assessment and laboratory blood analyses. BMI was determined by dividing body weight (kg) by the square of height (m^2^). Waist and hip circumferences, along with WHR, were measured using a standard clinical measuring tape. Blood pressure was assessed using the oscillometric method (OMRON 7320; São Paulo, SP, Brazil), following [24]. Biochemical analyses including cholesterol, HDL, LDL, insulin, glucose, and triglycerides were performed according to Da Silva, et al., 2025. The homeostasis model assessment of insulin resistance (HOMA‐IR) and β‐cell function (HOMA‐β) were calculated following previously established methods [25, 26].

### 2.3 Saliva Collection

Saliva samples were collected after a 12‐h fasting period and 1 h without liquid intake, as described in Da Silva et al., 2022 [27]. Saliva solution was collected into sterile tubes, and immediately mixed with 1 mM phenylmethylsulfonyl fluoride (PMSF; Sigma‐Aldrich, St. Louis, MO, USA), 1 mM ethylenediamine tetraacetic acid (EDTA), and 1 mM protease inhibitor cocktail (Sigma–Aldrich) to prevent protein degradation [28, 29].

Saliva samples were processed according to previously established protocols [29], with modifications. After centrifugation, the supernatant was concentrated using an Amicon Ultra Centrifugal Filters 3 kDa (Sigma–Aldrich) and were stored at −80°C to ensure sample integrity until further processing.

### 2.4 Protein Processing for Mass Spectrometry

All samples were processed uniformly using the following protocol. Protein quantification was performed using the Bradford assay, with absorbance measured on an Epoch Microplate Spectrophotometer (BioTek, Highland Park, USA). A calibration curve was generated using bovine serum albumin (BSA; Bio‐Rad Laboratories Inc., Hercules, CA, USA) as the standard [30].

For this work, a sample pooling strategy was adopted for proteomic analysis. Samples were grouped according to shared clinical characteristics, BMI, T2DM, and MetS, and categorized as follows: normal weight/control (*n* = 29), overweight (*n* = 25), obese (*n* = 15), MetS (*n* = 23), and T2DM (*n* = 11). This approach was designed to ensure homogeneity within each pool. To maximize donor representation while optimizing high‐resolution mass spectrometry throughput during the discovery phase, a tiered biological subpooling and analytical replication design was executed. To capture group‐specific molecular phenotypes while filtering individual confounding noise, each clinical cohort was partitioned into smaller, nonoverlapping biological subpools by combining equivalent protein concentrations from distinct subject donors, yielding 16 independent physical subpools across the clinical spectrum: normal weight 1 (*n* = 10), normal weight 2 (*n* = 10), normal weight 3 (*n* = 9); overweight 1 (*n* = 9), overweight 2 (*n* = 9), over‐weight 3 (*n* = 7); obese 1 (*n* = 5), obese 2 (*n* = 5), obese 3 (*n* = 5); MetS 1 (*n* = 8), MetS 2 (*n* = 8), MetS 3 (*n* = 7); T2DM 1 (*n* = 3), T2DM 2 (*n* = 3), T2DM 3 (*n* = 3), and T2DM 4 (*n* = 2). Each subgroup was constructed by combining equivalent protein concentrations from individual samples. This stratification was designed to balance biological representation and the distribution of key clinical characteristics across subgroups, thereby improving the generalizability of findings and minimizing potential biases. While this strategy inherently limits the assessment of interindividual variability, it provides a framework for Discovery phase proteomics, aiming to characterize the proteomic shifts and the host‐microbiota crosstalk landscape across metabolic states. Crucially, to explicitly monitor instrument drift and technical reproducibility, each of the 16 distinct biological subpools was processed and analyzed in strict technical triplicates via independent liquid chromatography‐tandem mass spectrometry (LC‐MS/MS) injections, resulting in a dense dataset of 48 analytical runs. For downstream computational processing, multi‐group Analysis of Variance (ANOVA), and the unbiased random forest (RF) classification framework, all 48 technical replicate runs were utilized simultaneously to preserve analytical variance and maximize cross‐group signal resolution.

Each pooled salivary proteome was subjected to trypsin digestion by using a shotgun method [31]. Briefly, 50 µg of each sample were denatured with 8 M urea (1:1 ratio), followed by reduction with 5 mM dithiothreitol (DTT) for 25 min at 56°C. To prevent protein modifications such as carbamylation of lysines and N‐terminal residues, temperatures above 60°C were avoided [34, 35, 36]. Alkylation was performed using 14 mM iodoacetamide for 30 min at room temperature in the dark. The urea concentration was diluted to 1.6 M with 60 mM ammonium bicarbonate, and 1 mM calcium chloride was added to the samples. Trypsin digestion was carried out using sequencing grade modified trypsin (Promega, Madison, WI, USA) at an enzyme to substrate ratio of 1:50. The reactions were incubated for 16 h at 37°C and terminated by adding 0.4% formic acid. To remove urea and other impurities, digested samples were purified using Oasis HLB 1cc columns (Waters Corporation, Milford, MA, USA).

### 2.5 Mass Spectrometry Analysis

All digested samples were analyzed as previously established protocols [21, 26, 29, 32]. They were subjected to direct injection into the mass spectrometer, processed sequentially for normalization, and analyzed in triplicate for quantitative assessment. Following proteomic data acquisition from saliva samples, a rigorous statistical analysis was conducted to identify significant variations in protein abundance across experimental groups. To enhance data reliability and mitigate technical variability, all three analytical replicates for each subgroup were included in the final analysis. In total, 48 samples were subjected to statistical evaluation, yielding a comprehensive dataset for robust statistical inference.

The injection sequence was designed to run one technical replicate of all of the samples each time, allowing sample integrity to be monitored throughout the analysis period. Blank samples (consisting of the sample resuspension solution) were injected periodically between different sample groups to avoid and monitor carryover effects. For normalization, each sample were analyzed in a preliminary LCMS run using an injection volume of 1 µL. In this run, the area of the total ion current (TIC) chromatograms were integrated, and the injection volume for subsequent analyses in replicates was adjusted so that the TIC areas of the different samples were within the same order of magnitude [37]. This strategy helped to ensure a comparable signal intensity between samples and reduce variability associated with differences in sample loading that could result in detector saturation. In addition, the software Progenesis QI for Proteomics (Nonlinear Dynamics, Waters Technologies, USA) was used to perform an internal normalization procedure to correct for systematic experimental variation. The default normalization option (normalize to all proteins) was applied. In this method, one run was automatically selected as the normalization reference, and abundance ratios between runs were calculated for all detected peptide ions. A global scaling factor was then estimated from these ratios and applied to each run to correct for systematic differences in signal intensity across different samples.

Following proteomic data acquisition from saliva samples, a rigorous statistical analysis was conducted to identify significant variations in protein abundance across experimental groups. To enhance data reliability and mitigate technical variability, all three analytical replicates for each subgroup were included in the final analysis. In total, 48 samples were subjected to statistical evaluation, yielding a comprehensive dataset for robust statistical inference.

Tryptic peptidomes were analyzed using a nanoUPLC system coupled to an electrospray quadrupole time of flight mass spectrometer (ESI‐Q‐TOF Synapt HDMS G1; Waters Corporation). Chromatographic separation was performed using a two‐phase system: mobile phase A consisted of 0.1% formic acid in water (v/v), while mobile phase B contained 0.1% formic acid in acetonitrile (v/v). Sample desalting was carried out on a nanoACQUITY UPLC Symmetry C18 Trap Column (5 µm, 20 mm, and 180 µm) at a flow rate of 5 µL/min for 3 min. Peptide separation was achieved using a nanoACQUITY UPLC HSS T3 reversed phase column (1.8 µm, 100 mm, and 100 µm) with a constant flow rate of 0.6 µL/min. The applied linear gradient was as follows: 7%−40% mobile phase B from 0 to 19.9 min, 40%−85% B from 19.9 to 23.9 min, 85% B maintained for an additional 4 min, followed by column reequilibration to initial conditions from 27.9 to 29.9 min, with an additional 7 min rinsing step.

Mass spectrometry was performed using a positive nano electrospray ionization (nanoESI+) source in MSe mode, employing data independent acquisition with parallel fragmentation of all precursor ions and alternating acquisition at low and high energy. At low energy, continuum spectra were recorded within a mass range of 50–2000 Da, with a scan time of 0.8 s. Instrument parameters included a source temperature of 80°C, a desolvation temperature of 100°C, a sampling cone voltage of 35 V, a capillary voltage of 3.0 kV, and a constant collision energy of 6 V. Under high energy conditions, all parameters remained unchanged except for the collision energy, which was ramped from 15 to 55 V throughout the scan. The mass spectrometer was calibrated using a 300 fmol/µL solution of human (Glu1)‐fibrinopeptide B (Waters Corporation), which served as a reference standard and was injected at a flow rate of 0.2 µL/min every 30 s. The acquired mass spectra were calibrated against the doubly charged precursor ion of (Glu1)‐fibrinopeptide B (785.8426 Da) to ensure high mass accuracy [24].

### 2.6 Statistical Analysis

To evaluate molecular shifts across the five clinical cohorts, data normality was rigorously tested within each group using the Shapiro–Wilk test (*p* > 0.05). A one‐way Analysis of Variance (ANOVA) was executed to determine statistically significant differences across multiple groups. To avoid inflation of Type I errors, the false discovery rate (FDR) method was applied for multiple testing corrections (*q* < 0.05). The magnitude of differences was quantified by reporting effect sizes via Eta‐squared (*η* ^2^), accompanied by their respective 95% confidence intervals (CIs) computed using a stratified bootstrapping approach with 1000 resamples. To accommodate the distinct biological layers of our study design and prevent excessive statistical conservatism, multi‐group univariated *p*‐values were adjusted utilizing the structure‐adaptive Benjamini–Hochberg Algorithm (SABHA) at a target global false discovery rate (FDR). Nonparametric alternatives were strictly restricted to variables failing normality criteria. Permutational multivariate analysis of variance (PERMANOVA) and homogeneity of multivariate dispersions (PERMDISP) tests were implemented to assess global expression boundaries and evaluate within‐group structural variability across the five continuous clinical groups. Both analyses were executed based on Euclidean distance matrices using a permutation‐based approach, 999 iterations [33]. Due to the sample pooling experimental framework, technical drops and individual biological variance were highly controlled. To address potential left‐censoring and low‐abundance phenomena, these sparse missing values were categorized as missing not at random (MNAR) and imputed using the quantile regression imputation of left‐censored data (QRILC) framework, which fits a truncated log‐normal distribution to the lower tail of the dataset. A comparative sensitivity analysis was subsequently performed by calculating and correlating the multi‐group analysis of variance (ANOVA) *F*‐statistics, *p*‐values, and Eta‐squared effect sizes derived from the complete case dataset against the QRILC‐imputed dataset to evaluate pipeline stability.

Mass spectrometry (MS) data were processed and searched against the UNIPROT database, which contains 20,379 human proteins (2018), including only reviewed entries (https://www.uniprot.org/), using the Progenesis QI 4.06 software (Waters). To identify bacterial proteins, MS data were also searched against a database of eight bacterial phyla (Actinobacteria, Bacteroidetes, Chloroflexi, Chlamydiae, Fusobacteria, Firmicutes, Synergistetes, and Spirochaetes) from UNIPROT, with 161.685 proteins. To avoid false‐positive peptide identifications, trypsin specificity was enforced during the database search.

Peptide and protein identifications were performed using Progenesis QI for Proteomics, following the manufacturer's recommended stringency parameters for MSE data processing. The search criteria were set to require a minimum of 3 fragment ion matches per peptide, seven fragment ion matches per protein, and at least one unique peptide match per protein [38, 39, 40]. One missed trypsin cleavage was allowed, with carbamidomethylation of cysteines as a fixed modification and oxidation of methionine as a variable modification [29]. Proteins were considered identified only if they matched at least one unique and specific peptide from the experimental data. Protein abundance was estimated based on the average abundance of the three most intense tryptic peptides [33, 35]. To ensure the highest level of identification confidence and minimize false discoveries, identifications were classified as valid if detected in at least two analytical replicates, with a false discovery rate (FDR) of <1% across all data.

Quantitative analysis was performed on proteins identified in at least 50% of the samples within at least one group. This filtering threshold was adopted to balance proteome coverage with statistical power, ensuring that proteins with biological relevance in specific pathological states were not prematurely excluded due to the high inter individual variability inherent in clinical metabolic studies. Following established proteomics workflows [41, 42], missing values were not imputed to avoid the introduction of artificial noise or potential inflation of false positives; instead, statistical comparisons were conducted using only experimentally measured intensities.

Quantitative variances were assessed using ANOVA in the MetaboAnalyst 6.0 platform (https://www.metaboanalyst.ca/home.xhtml), with a significance threshold of *p* ≤ 0.05. Partial least squares discriminant analysis (PLS‐DA) and Pattern Hunter (PH) were employed to evaluate group differences. Pearson's correlation analysis was per‐formed using PH, with correlations classified as moderate (0.5–0.7), high (0.7–0.9), or very high (0.9–1.0) [43]. Protein clinical data correlations were visualized using the corrplot package (Version 0.92) [44]. To identify key features differentiating groups, two multivariate classification algorithms were applied: PLS‐DA, used to calculate the variable importance in projection (VIP) score, and RF. The VIP score quantifies the contribution of each variable to the PLS‐DA model, with scores ≥1 considered significant [45]. To validate the discriminative power of the supervised models (random forest and PLS‐DA) and address potential overfitting risks, we conducted a PERMANOVA analysis with 999 permutations based on Bray–Curtis distances. RF, a machine learning algorithm, was used to rank protein features based on their relevance for sample classification [46], and to provide predictive insights into disease risk based on patient characteristics [47, 48]. The reliability of RF results was confirmed using receiver operating characteristic (ROC) curves, confusion matrices, and error rates, generated for both human and bacterial proteins using the mixOmics package (Version 6.26) [49].

Given that PLS‐DA is highly supervised and inherently prone to capturing random noise in large scale omics designs, the statistical significance of the optimal model was validated using a rigorous permutation test. An empirical *p*‐value was calculated by comparing the predictive performance of the original unpermuted model against the distribution of the 1000 permuted models. A threshold of an FDR‐corrected *p* < 0.05 was established, confirming that the observed group separation was not a product of random chance or data artifacts. To identify the key molecular features driving the phenotypic discrimination between the experimental groups, the variable importance in projection (VIP) scores were calculated for each variable across the validated components. The VIP score represents a weighted sum of squares of the PLS loadings, reflecting both the amount of explained Y‐variance and the correlation between the feature and the biological groups. Features with a VIP score > 1.0 were considered highly stable and significant contributors to the model's discriminatory power. The top‐ranking features were further inspected across cross‐validation folds to ensure selection stability and minimize false discovery rates.

To determine the optimal number of latent variables (components) and prevent overfitting, a cross‐validation (CV) algorithm was employed. Model performance was systematically evaluated using three complementary metrics: a) accuracy; b) Goodness‐of‐fit R2; c) Goodness‐of‐prediction Q2.

To rigorously evaluate the statistical trade‐offs of the experimental design and assess the impact of sample pooling on data variance, a post‐hoc power analysis was conducted using the Experimental Design module of MetaboAnalyst 6.0. Empirical power curves were simulated to compare the statistical behavior of the current pooled framework against a projected individual sampling design. The mathematical parameters of the simulations were derived directly from the experimental multi‐group dataset. Monte Carlo simulations were executed across varying sample sizes to project the predicted statistical power after applying a false discovery rate (FDR) correction threshold of 0.05.

## 3 Results

### 3.1 Demographic and Clinical Evaluation

As described in Ferreira da Silva, 2024, analysis of clinical parameters revealed significant changes associated with increasing BMI and the presence of MetS and T2DM (Table S1). A notable increase in systolic blood pressure was observed in the overweight group. Interestingly, insulin levels significantly differed between control groups (NW, OW) and MetS, but not in relation to the T2DM. In individuals with type 2 Diabetes Mellitus (T2DM), peripheral insulin resistance initially leads to a compensatory increase in insulin secretion by pancreatic β‐cells [48]. This heightened demand imposes functional overload on β‐cells, triggering endoplasmic reticulum stress, oxidative stress, mitochondrial dysfunction, and loss of cellular identity. Over time, these mechanisms promote activation of proapoptotic pathways, resulting in reduced β‐cell mass and function. Consequently, there is a progressive decline in insulin production, contributing to the relative insulin deficiency characteristic of T2DM [50, 51].

### 3.2 Metaproteome Profiling of Saliva

The human and bacterial salivary proteomes were analyzed using quantitative mass spectrometry (LC‐ESI Q/TOF) with a gel independent approach. This method enabled the identification of 66 human proteins (Table S2), of which 35 exhibited differential abundances across the study groups. One hundred and twenty one bacterial proteins were identified (Table S3), with 55 showing differential expression when comparing all groups. Following a significant ANOVA result, pairwise comparisons of protein abundances between groups were conducted using a post hoc Tukey's HSD test and hierarchical SABHA for FDR (Table S2 and S3).

The taxonomic classification of the 55 differentially expressed bacterial proteins revealed a predominance of members from the phyla Firmicutes (12 proteins), Chloroflexi (11), Fusobacteria (10), and Chlamydiae and Actinobacteria (7 each). At a finer taxonomic level, the most represented orders were Fusobacteriales and Bacillales (10 proteins each), followed by Chloroflexales (8) and Chlamydiales (7), reflecting the dynamic composition of taxa typically associated with mucosal environments and host–microbiota interactions [52, 53] (Table S4). In terms of expression patterns, Firmicutes exhibited eight upregulated and four downregulated proteins, Chloroflexi showed seven downregulated and four upregulated, Fusobacteria had seven up‐regulated and three downregulated, and Chlamydiae displayed six upregulated and one downregulated protein. Consistently, at the order level, Fusobacteriales presented seven upregulated and three downregulated proteins, Bacillales had six upregulated and four downregulated, Chloroflexales exhibited six downregulated and two upregulated, and Chlamydiales showed six upregulated and one downregulated, reinforcing the heterogeneous regulation among bacterial taxa under the studied condition.

The sample pooling framework successfully controlled technical dropouts, yielding near perfect completion rates of 99.76% for the human saliva proteome (four missing values out of 1680) and 99.70% for the bacterial secreted metaproteome (eight missing values out of 2640, restricted to features B8G5×3 and B1YI73). To address potential low‐abundance artifacts, these sparse entries were classified as MNAR and imputed using the (QRILC) framework, which models values from the lower tail of the distribution. The analysis demonstrated an absolute statistical convergence across both workflows, with Pearson correlation coefficients exceeding *r* = 0.999999 for all metrics. For instance, the human proteome size of effect for P69905 varied negligibly from *η* ^2^ = 0.5011 (complete case) to *η* ^2^ = 0.5012 (imputed), while for Q9UP65 it remained completely stable (*η* ^2^ = 0.2980). For bacterial proteins the effect size for B8G5×3 remained fixed at *η* ^2^ = 0.3810 in both pipelines, while for B1YI73 it was entirely stable at *η* ^2^ = 0.2423.

**FIGURE 2:**
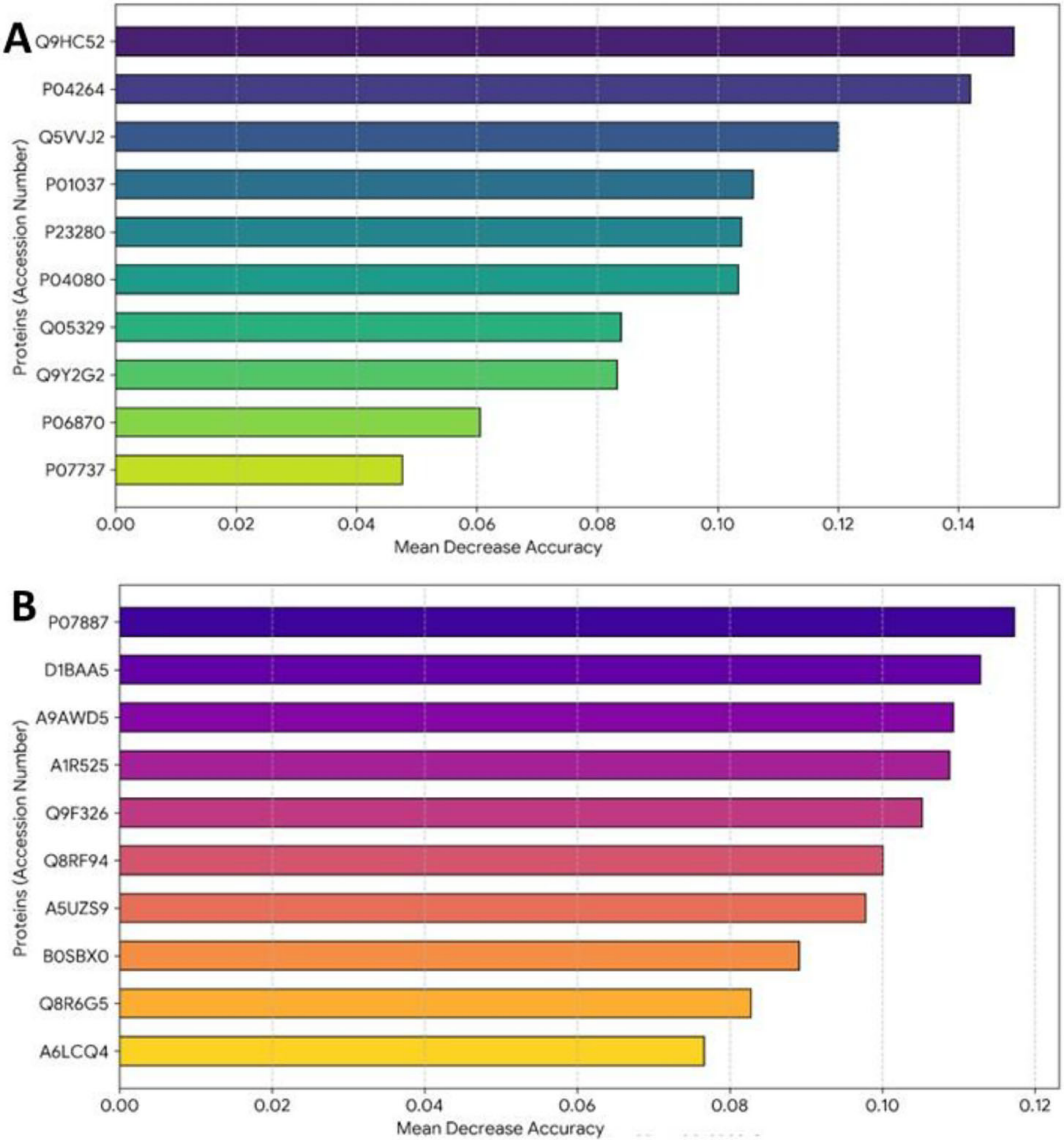
Random Forest Analysis of Top 10 Human and Bacterial Proteins (Genes names). Panel (A) displays the mean decrease in accuracy (MDA) plot for human proteins. Similarly, panel (B) presents the MDA plot for bacterial protein profiles.

The exploratory RF analysis, managed through subpool‐stratified cross‐validation to account for technical variance dependencies, highlights a suggestive panel of top‐ranking candidates, proposing them as prioritized leads for metabolic stratification. A RF was employed to generate the top 10 proteins (Figures 2A and 2B; Table S5), including Deubiquitinase MYSM1 (*η* ^2^ = 0.710, adjusted q 1.9 e‐11), Carbonic anhydrase 6 (*η* ^2^ = 0.374, adjusted 4.2e‐05), and Glutamate decarboxylase 2 (*η* ^2^ = 0.478, adjusted q1.6e‐06) for the human host proteins. Concurrently, core features from the bacterial secreted metaproteome fraction stood out, such as Ribosomal RNA small subunit methyltransferase H (*η* ^2^ = 0.772, adjusted q 1.4e‐13), uridylate kinase (*η* ^2^ = 0.629, adjusted q1.3e‐09), and Thioredoxin C‐2 (*η* ^2^ = 0.557, adjusted q 5.0e‐08). These molecular characteristics potentially reflect the metabolic change observed with increased BMI and the presence of MetS and T2DM. For the human host salivary proteomic, the model yielded a strong classification profile, displaying a OOB error rate of 0.1875 (18.75%). Concurrently, the bacterial metaproteomic secreted model demonstrated a slightly superior classification performance, achieving a OOB error rate of 0.1458 (14.58%). Additionally, variable importance in projection (VIP) Score analysis was conducted to identify the key classifying proteins among the studied groups (Figure S1A,B).

The multivariate model revealed a robust and significant separation between the metabolic phenotypes for both human salivary proteins (*F* = 16.345, R2 = 0.603, and *p* = 0.001) and bacterial proteins (*F* = 18.675, R2 = 0.634, and *p* = 0.001). The high R2 values (Tables S6) demonstrate that the clinical classification (NW, OW, OB, MetS, and T2DM) accounts for more than 60% of the proteomic variance, indicating a strong biological signal that outweighs technical or individual noise. Furthermore, the empirical *p*‐values derived from permutation testing confirm that the observed group clusters are statistically independent of random sample distribution. These findings provide a high degree of confidence, ensuring that the identified signatures are representative of the pathological state of metabolic disorders.

To confirm the structural segregation of the host and microbial profiles across the metabolic progression, global distance and variance architectures were quantified via PERMANOVA and PERMDISP testing. For the human host salivary proteome, the permutation framework revealed a highly significant multi‐group location shift (Table S7), alongside noticeable variations in internal biological variance across the cohorts (PERMDISP *F* = 5.6511; *p* = 0.001). Parallelly, the bacterial secreted metaproteome demonstrated pronounced variations, yielding a robust centroid separation (Table S7) accompanied by multivariate dispersions (PERMDISP *F* = 4.4435; *p* = 0.001).

The prediction model generated by RF demonstrated higher accuracy in classifying the study groups compared to the VIP Score analysis (Tables S7). To further validate the accuracy of the RF model, ROC curves were constructed (Figure 2A,B), and confusion matrices along with error rates were generated for both human and bacterial proteins (Tables S8 and S9).

**FIGURE 3:**
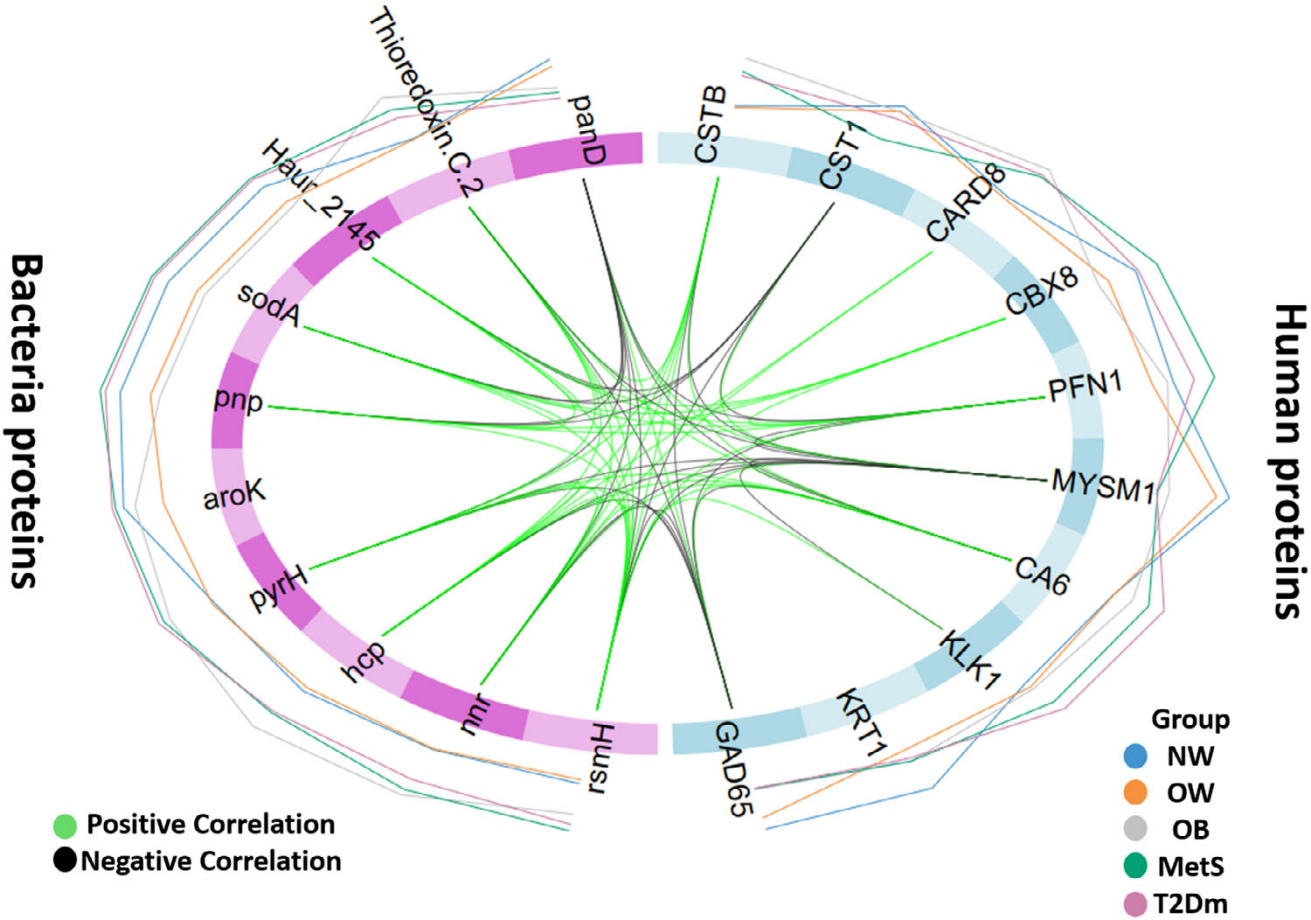
The figure illustrates the interactome analysis between the top 10 human and bacterial proteins. The Circos plot visually represents the interactions between human and bacterial proteins, with green lines indicating positive correlations and black lines denoting negative correlations.

The top 10 human and bacterial proteins selected by RF were used to create partial least squares‐discriminant analysis (PLS‐DA) plots (Figure 3A,B). Given human salivary proteins there was an effective distinction of the normal weight/control and overweight groups from the other three group. In contrast, the obese, MetS, and T2DM groups exhibited substantial overlap, indicating a higher degree of similarity in their salivary proteomes. Bacterial protein profiles also demonstrated a strong capacity to differentiate the study groups, with some overlap observed between the normal weight/control and overweight groups, as well as between the T2DM and MetS groups. Notably, OW samples showed specific data composition in terms of bacterial proteins in saliva.

To evaluate the multi‐group separation across the five clinical cohorts, PLS‐DA models were validated for both datasets. The human salivary proteomic cross‐validation (Figure S4A) showed a two component model, sustaining an Q2 score of 0.654 and an R2 score of 0.751 (Accuracy = 0.531). For the bacterial metaproteomic, cross‐validation (Figure S4B) selected a 2‐component architecture as the optimal model, achieving a Q2 of 0.611 and a R of 0.740 (Accuracy = 0.50). Permutation tests (1000 iterations) were executed to assess the risk of overfitting and confirm that the multi‐group separations were not driven by random noise. For the human salivary proteins (Figure S5A), the empirical permutation test confirmed high statistical significance, yielding a *p*‐value of < 0.001 (0/1000), as the observed cross‐validated statistic completely separated from the random background distribution. Consistently, the bacterial metaproteomic secreted (Figure S5B) also demonstrated significance with a permutation *p*‐value of < 0.001 (0/1000).

A prospective power analysis was performed across simulated cohort sizes to validate the expe. The injection sequence was designed to run one technical replicate of all of the samples rimental design and evaluate the impact of sample pooling on statistical sensitivity. As shown in the newly generated power profiles (Figure S6), the empirical power curve confirms that the individual design (Figure S6A) retains higher statistical power across identical sample sizes compared to the pooling design, human and bacterial proteins (Figure S6B,C). For instance, at a projected sample size of *n* = 100 per group, the predicted power increases from 0.21 and 0.18 in the pooled model, human and bacterial proteins respectively, to 0.38 in the individual framework.

### 3.3 Integrative Analysis of Clinical and Metaproteome Data

For analysis of interactions between the bacterial and human proteomes, as well as between the metaproteome and clinical data, the mixOmics and corplot packages were utilized. The corplot package was employed to assess correlations between human and bacterial proteins with clinical data (Tables S10, S11, and S12).

Among human proteins, Kallikrein‐1 (Table S10) and carbonic anhydrase 6 exhibited strong and direct correlations with systolic blood pressure and fasting glucose levels. Conversely, Deubiquitinase MYSM1 and glutamate decarbox‐ylase two displayed negative correlations with body mass, BMI, waist circumference, hip circumference, WHR, systolic blood pressure, and diastolic blood pressure. Deubiquitinase MYSM1 was negatively correlated with insulin levels, HOMA‐β, and HOMA‐IR.

In bacterial proteins, thioredoxin C‐2 (Table S11), Hydroxylamine reductase, Ribosomal RNA small subunit methyltransferase H, and uridylate kinase demonstrated positive correlations with BMI, waist circumference, hip circumference, and WHR. Furthermore, ribosomal RNA small subunit methyltransferase H and uridylate kinase were positively correlated with HOMA‐IR and negatively correlated with HDL levels.

The investigation of human and bacterial proteins correlation, and their potential impact on individual health, revealed significant interactions among them. Specifically, the bacterial thioredoxin C‐2, hydroxylamine reductase, ribosomal RNA small subunit methyltransferase H and uridylate kinase exhibited a strong negative correlation with the human protein Deubiquitinase MYSM1 and a moderate negative correlation with glutamate decarboxylase 2 (Figure 3). In contrast, the human protein Carbonic anhydrase 6 (Table S12) demonstrated a positive correlation with the aforementioned bacterial proteins (thioredoxin C‐2, hydroxylamine reductase, ribosomal RNA small subunit methyltransferase H, and uridylate kinase).

To robustly quantify the magnitude of molecular variations across the five clinical cohorts (NW, OW, OB, MetS, and T2DM) and accommodate potential non normal distribution patterns, an extensive effect size analysis using Eta‐squared (*η* ^2^) was executed. To mathematically rule out cohort‐specific artifacts and evaluate the precision of these metrics under the constraints of sample pooling, their respective 95% CIs were empirically derived using a stratified bootstrapping framework (1000 resamples per cohort) for both the human host salivary proteome and the bacterial metaproteome (Tables S13 and S14).

The 10 human and bacterial proteins selected by RF were analyzed using the Hunter Pattern method. Pearson's correlation analysis was conducted via PH to assess the relationships between these proteins and increased BMI, as well as the presence of T2DM and MetS. The results for human proteins are presented in Figure S7A and Table S15, while bacterial protein correlations are shown in Figure S7B and Table S15. Among the identified human proteins, Deubiquitinase MYSM1, carbonic anhydrase 6, and glutamate decarboxylase 2 exhibited significant correlations with increased BMI and the presence of MetS and T2DM. Similarly, the bacterial proteins uridylate kinase, aspartate 1‐decarboxylase, and the bifunctional NAD(P)H‐hydrate repair enzyme Nnr demonstrated significant correlations with high BMI and the studied diseases.

**FIGURE 4:**
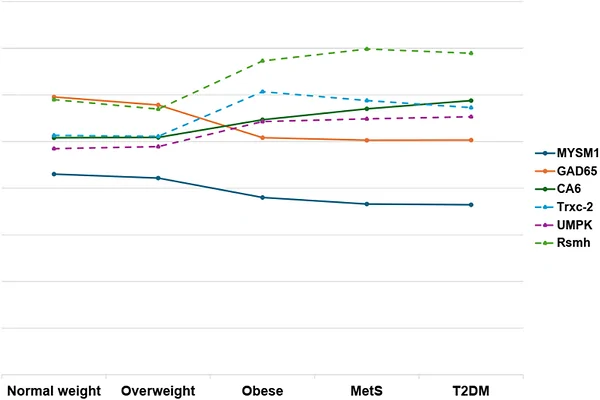
This diagram depicts the intricate network of interactions between human (solid lines) and bacterial (dashed lines) proteins, and how these connections relate to obesity, metabolic syndrome, and type 2 diabetes. The visualization underscores the multifactorial nature of these conditions, highlighting the potential roles and interdependencies of diverse protein profiles. All values were logarithmically transformed to improve comparability and facilitate visualization across variables measured in different units.

To provide a comprehensive overview of the host–microbiota interactome, we proposed an integrated biological model (Figure 4). This model synthesizes the proteomic shifts observed across the clinical groups.

## 4 Discussion

In this study, we aimed to perform an integrative analysis of the human and bacterial proteins in the saliva supernatant metaproteome from Brazilian volunteers, an approach that has been little explored in the literature, particularly within the context of the oral cavity. The proteomic coverage reflects the analytical capacity of the Synapt G1 platform using MS^E^ acquisition. Furthermore, the metaproteomic analyses did not include processing steps for enrichment of bacterial cells. We focused on identifying high‐confidence, representative proteins that characterize the dominant biological shifts in metabolic disorders. This approach allowed us to identify significant relationships between human and bacterial proteins during increased BMI and the presence of MetS and T2DM conditions. Furthermore, our findings are consistent with previous salivary proteomics studies, which have reported protein alterations associated with increased BMI and the presence of MetS and T2DM [21, 22, 27].

Among the differentially expressed bacterial proteins, twelve were identified as belonging to the phylum Firmicutes, which includes the order Bacillales. In a study conducted in an Iraqi population with normal, overweight, and obese categories, Firmicutes were found to be significantly more abundant in the oral microbiome as BMI increased [54]. Conversely, a study conducted with a Rio de Janeiro population, comparing individuals across BMI categories and with MetS, observed a decrease in the relative abundance of Firmicutes from 33.7% in the control group to 30.4% in the MetS group [55]. Although the second study did not reveal statistically significant differences, the contrasting outcomes observed may reflect the multifactorial nature of the oral microbiota, which is strongly influenced by metabolic disorders, geographic location, dietary habits, and the host's physiological status. Notably, proteins assigned to the phylum Firmicutes, particularly those from the order Bacillales, exhibited a general trend toward increased expression, including the superoxide dismutase [Mn/Fe] (Q9F326) from Staphylococcus, that was assigned as an important component to differentiate diseased groups and was higher in these last groups. It suggests that members of this taxon may play an active role in modulating oral microbial dynamics under the studied conditions.

The phylum Chloroflexi presented eleven differentially expressed proteins, with eight assigned to the order Chloroflexales. Notably, a previous study of the subgingival microbiome in Chilean patients identified Chloroflexi in both healthy individuals and those with chronic periodontitis, showing a higher relative abundance in diseased subjects [56]. This observation supports the potential involvement of Chloroflexi in oral inflammatory processes, although their specific contribution to the pathogenesis of periodontal disease remains to be elucidated. In the present analysis, most Chloroflexi proteins exhibited decreased expression. However, three of the upregulated proteins were pointed in the top 10 RF biological markers for MetS and T2DM, and specially Ribosomal RNA small subunit methyltransferase H, that correlated positively with BMI and disease status.

The phylum Fusobacteria, represented primarily by the order Fusobacteriales, was identified among the differentially expressed bacterial proteins in our analysis. Within this group, *Fusobacterium nucleatum* is particularly notable for its role as a key bridging organism in oral biofilms, mediating intergeneric coaggregation between obligate anaerobes and oxygen tolerant species, thereby facilitating biofilm maturation and stability [57, 58]. Beyond its local effects, *F. nucleatum* has been implicated in the pathogenesis of systemic conditions, including cardiovascular disease, cancer, inflammatory bowel disease, respiratory infections, Alzheimer's disease, and rheumatoid arthritis [59]. Recent studies have also reported an increased abundance of the Fusobacterium genus in individuals with metabolic syndrome [17, 55]. Consistent with these findings, our results revealed that most Fusobacteria proteins were upregulated in association with weight gain and the presence of MetS and T2DM, suggesting a potential link between the metabolic state of the host and the functional activity of Fusobacteria in the oral microbiome.

**FIGURE 5:**
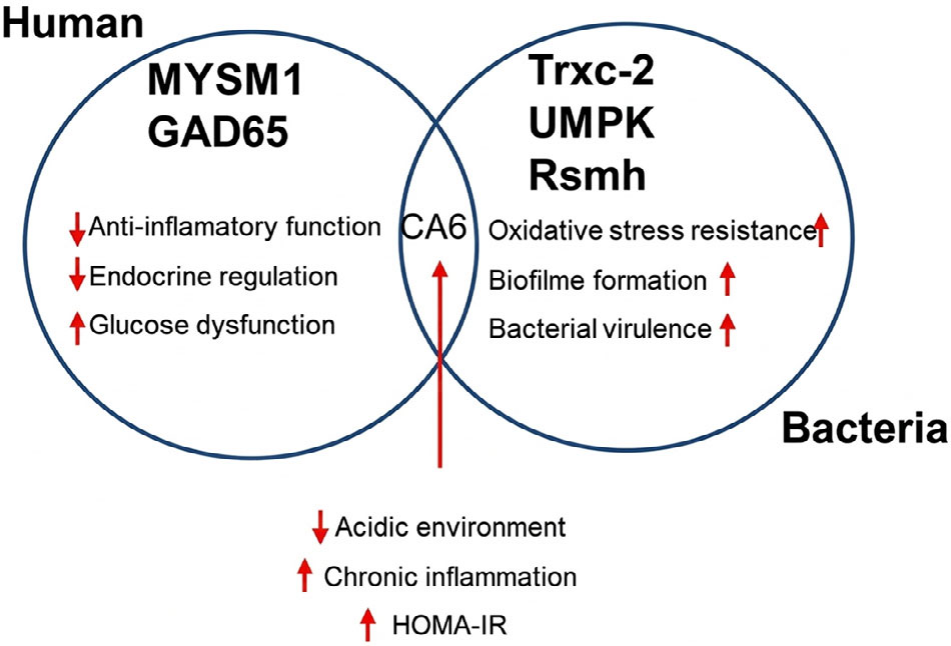
Interaction between human and bacterial proteins associated with metabolic disorders. The diagram illustrates the functional synergy between human (MYSM1, GAD65) and bacterial (TrxC‐2, UMPK, RsmH) proteins, highlighting the CA6 protein as the intersection point. Decreased human proteins are associated with glycemic dysfunction and inflammation, while increased bacterial proteins contribute to oxidative stress and microbial virulence. The mutual interaction, mediated by an acidic environment, promotes chronic inflammation and is correlated with increased insulin resistance (HOMA‐IR).

Overall, our results revealed some positive and negative correlations between human and bacterial proteins with clinical data, providing insights about host‐oral microbiome interaction during these pathological processes of obesity (Figure 5).

**FIGURE 6:**
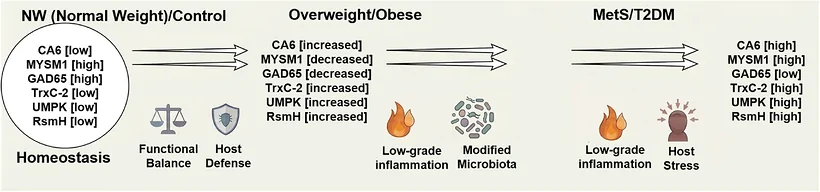
Progression of metabolic dysregulation and microbial modulation across body weight gain and clinical status. The diagram illustrates the transition from homeostasis in normal weight (NW) individuals to states of overweight/obesity and metabolic syndrome/type 2 diabetes mellitus (MetS/T2DM).

The deubiquitinase protein MYSM1 is a metalloprotease with critical roles in hematopoietic stem cells, lymphocytes, repair of DNA damage, mature blood cells and protective role against excessive inflammation, and autoimmune reactions [60, 61]. MYSM1 has also been shown to exhibit a protective effect against colorectal cancer. In mice, the deletion of MYSM1 resulted in a higher mortality rate following viral infections, while treatment with MYSM1 demonstrated the ability to suppress proinflammatory cytokines [61, 62]. Despite scarce literature linking MYSM1 to metabolic disorders, our data reveal a robust inverse association between MYSM1 levels and both BMI and disease status. MYSM1 abundance declined by more than 1.7‐fold in obese individuals and by over 2‐fold in subjects with MetS and T2DM (Figures 5 and 6) compared to normal‐weight controls (NW). Additionally, MYSM1 exhibited a strong negative correlation with waist circumference and WHR (Tables S11 and S12). Furthermore, MYSM1 showed significant negative correlations with thioredoxin C‐2, hydroxylamine reductase, ribosomal RNA small subunit methyl‐transferase H, and uridylate kinase. Although the role of MYSM1 in bacterial modulation remains poorly understood, our findings reveal a strong negative correlation between MYSM1 and bacterial proteins associated with oxidative stress resistance. This suggests that MYSM1 may be associated with regulatory role in some members of the salivary microbiota.

Glutamate decarboxylase (GAD65) is an enzyme expressed in neurons and pancreatic beta cells, where it catalyzes the conversion of glutamate to γ‐aminobutyric acid (GABA) [63]. GABA is synthesized not only by pancreatic β cells but also by immune cells and intestinal bacteria, having broad physiological relevance [64]. Within the pancreas, GABA produced by GAD65 plays a crucial role in modulating islet function, including the suppression of glucagon secretion by α cells and the promotion of β cell survival [65]. GAD65 is also implicated in the pathogenesis of type 1 diabetes mellitus (T1DM), as it is a major autoantigen targeted by the immune system in this condition [66]. In patients with T1DM, the presence of autoantibodies against GAD65 is a hallmark of the autoimmune destruction of β cells [67, 68]. While the role of GAD65 in T1DM is well established, its relationship with other metabolic diseases, such as obesity, MetS, and T2D, remains poorly understood. In this study, GAD65 levels were significantly reduced (Figures 5 and 6) in these three groups (by more than 2.5‐fold decrease in obese, MetS and T2DM) compared to normal‐weight/control and overweight individuals (Table S10). Additionally, GAD65 exhibited negative correlations with body mass, systolic and diastolic blood pressure, HOMA‐IR, WHR, waist, and hip circumference (Table S10). The reduction in GAD65 levels in individuals with high BMI, MetS, and T2DM, along with its inverse associations with clinical and anthropometric obesity parameters, suggest that GAD65 may be associated with a lower risk of developing metabolic disorders and underscores the need for further research to elucidate the possible mechanisms linking GAD65 to these conditions.

Carbonic anhydrases (CAs) are metalloenzymes classified as a superfamily comprising eight families to date, including two cytosolic forms, four membranes bound, one mitochondrial, one secreted form, and carbonic anhydrase VI [69]. CAs are involved in proton generation by catalyzing the reversible hydration of carbon dioxide (CO_2_ + H_2_O ⇌ H^+^ + HCO_3_^−^), with increased CA expression being associated with higher concentrations of H^+^ and HCO_3_^−^ ions, leading to local extracellular acidosis [70]. Studies have reported an inverse correlation between the intensity of inflammation, as seen in rheumatoid arthritis, and pH levels, implicating CAs in several inflammatory processes [70]. Obesity, MetS, and T2DM are metabolic diseases closely linked to chronic low‐grade inflammation [72]. Lamy et al. [72] observed elevated levels of CA 6 in individuals with morbid obesity compared to both normal‐weight/control individuals and obese individuals who underwent bariatric surgery. In our study, CA 6 levels were more than 1.5 times higher in obesity (Figures 5 and 6) compared to the normal‐weight/control group, and more than 2 times higher in the MetS and T2DM groups. Elevated CA 6 levels are strongly and directly correlated with systolic blood pressure, blood glucose, and HOMA‐IR (Table S10), and showed positive correlations with bacterial proteins such as hydroxylamine reductase, RsmH, and uridylate kinase. These findings indicate that CA 6 overexpression in the oral cavity may promote an acidic and inflammatory microenvironment, and may therefore be associated with the development of these metabolic diseases, potentially serving as a key link between the microbiota, inflammation.

Alterations in the microbiota, or dysbiosis, are routinely observed in metabolic diseases such as obesity, MetS, and T2DM [17, 48]. Although the literature primarily focuses on changes in the diversity and abundance of the oral microbiota, studies on the metaproteome have gained prominence for their ability to elucidate interactions between the microbiota and the host [22]. In this work, several bacterial proteins, including Thioredoxin C‐2, Hydroxylamine reductase, Ribosomal RNA small subunit methyltransferase H, and uridylate kinase presented strong and inverse correlation with human proteins GAD65 and MYSM1 levels.

Uridylate kinase (also known as uridine monophosphate kinase, UMPK) is an evolutionarily conserved enzyme that catalyzes the reversible phosphorylation of UMP to UDP and subsequently to UTP [73]. The resulting UTP is critical for the synthesis of UDPN‐acetylmuramic acid, peptidoglycan, and RNA [73]. Recent studies have highlighted UMPK's role in virulence and biofilm production, directly influencing the relationship between the microbiota and its host. Notably, 4‐methoxy‐1‐methyl‐2‐oxopyridine‐3‐carbamide (MMOXC), a ricinin derivative, exhibits antibiofilm properties by inhibiting UMPK, thereby disrupting cell wall formation, RNA biosynthesis, and protein maturation [73, 74]. Emeka et al. [74] reported that UMPK expression was elevated nearly twofold in biofilms. In our study, UMPK expression showed a strong positive correlation with weight gain (Figures 5 and 6) and the presence of metabolic diseases (Table S11), with a greater than two‐fold increase from the normal‐weight/control (NW) group to the MetS and T2DM. UMPK also showed a strong and positive correlation with waist and moderate with mass, BMI, diastolic blood pressure and HOMA.IR. These findings suggest that elevated BMI and metabolic diseases conditions may facilitate bacterial colonization and maybe enhance the virulence of certain species, influencing the dynamics between the oral microbiome and host immune responses.

Ribosomal RNA small subunit methyltransferase H (RsmH, also known as MraW) is a bacterial Sadenosylmethionine–dependent methyltransferase that specifically methylates cytosine 1402 (C1402) of 16S rRNA, a modification essential for fine tuning the ribosomal decoding center and ensuring high fidelity and efficient translation [75, 76]. Beyond rRNA, rsmH can methylate DNA, thereby modulating expression of genes involved in motility and other cellular processes [77]. In Staphylococcus aureus, RsmH enhances resistance to oxidative stress crucial for surviving to host immune attacks. Zou et al. [71, 72, 73, 74, 75, 76, 77, 78] showed that a rsmH knockout reduces gentamicin tolerance by over tenfold in vitro. In *Escherichia coli*, rsmH also contributes to virulence by regulating flagellar synthesis and intestinal colonization [78]. In our study, rsmH abundance rose more than threefold in individuals with MetS and T2DM and over twofold (Figures 5 and 6) in obese subjects versus normal‐weight controls, correlating positively with BMI and disease status (Table S11). It also showed positive associations with waist circumference, WHR, diastolic blood pressure, fasting insulin, and HOMA‐IR, while correlating negatively with deubiquitinase MYSM1 and glutamate decarboxylase 2 and positively with carbonic anhydrase 6 (Table S12). These results suggest that rsmH's role in enhancing bacterial virulence and oxidative‐stress tolerance may drive oral microbiome dysbiosis, linked to obesity, MetS, and T2DM, and associated chronic inflammation, potentially involving carbonic anhydrase 6 pathways.

The thioredoxin system is a critical antioxidant defense mechanism conserved across diverse organisms, from bacteria to mammals [79]. Comprising thioredoxin (Trx), thioredoxin reductase (TrxR/TrxB), and nicotinamide adenine dinucleotide phosphate (NADPH), this system regulates cellular redox homeostasis. Thioredoxin, including isoforms such as thioredoxin‐A (TrxA) and thioredoxin‐C (TrxC), is a 12‐kDa thiol‐disulfide oxidoreductase with multifaceted roles. These include reducing protein disulfides, modulating key regulators of motility, enhancing pathogen adhesion to epithelial cells, and influencing virulence factors [80]. Beyond its enzymatic functions, the thioredoxin system is integral to DNA synthesis, gene transcription, cell growth, apoptosis, and protection against oxidative stress [81]. Recent studies highlight its pivotal role in stress resistance, motility, and microbial virulence, underscoring its relevance in both physiological and pathological contexts [82]. In our study, Thioredoxin C‐2 (TrxC‐2) was significantly upregulated in obese, MetS, and T2DM groups compared to normal‐weight/control and overweight controls (Figures 5 and 6). Specifically, TrxC‐2 levels in the obese group were over three times higher than in NW and OW individuals, while in the MetS and T2DM groups, levels were approximately doubled. Additionally, TrxC‐2 exhibited a moderate positive correlation with carbonic anhydrase 6. Conversely, TrxC‐2 showed a moderate negative correlation with glutamic acid decarboxylase 65 (Gad65) and a strong negative correlation with deubiquitinase MYSM1. The moderate positive correlations observed between TrxC‐2 and BMI, waist circumference, hip circumference, WHR, diastolic blood pressure, and insulin levels further indicate that TrxC‐2 upregulation may be linked to metabolic dysregulation and insulin resistance, hallmarks of obesity, MetS, and T2DM. These findings suggest a potential compensatory response to heightened oxidative stress associated with obesity and metabolic disorders, consistent with the thioredoxin system's role in mitigating reactive oxygen species (ROS) accumulation [83].

The biological magnitude of the observed protein shifts provides a translational framework for understanding host–microbiome interactions in metabolic health. The alterations in carbonic anhydrase VI (CA6) levels observed here align with previous reports linking CA6 to obesity related changes in taste sensitivity and oral homeostasis [72, 84]. However, both studies reported directional changes (increases or decreases) rather than quantifying the magnitude of these alterations. Notably, the fold changes reported for our novel candidates, such as MYSM1, GAD65, and specific bacterial stress proteins, are consistent with the recent high depth salivary study by Samodova et al. (2025), who reported effect sizes ranging from 0.19 to 1.9 in T2DM cohorts. The fact that these proteins exhibit robust statistical significance despite being reported here for the first time in a metabolic context suggests they represent sensitive indicators of systemic metabolic stress. These magnitudes are within the expected range for salivary biomarkers, where the protein matrix is inherently more diluted than plasma but reflects localized and systemic physiological shifts with high fidelity.

Recent studies can yield thousands of identifications in saliva samples, by using recent advanced mass spectrometry, such as ion mobility coupled systems (e.g., timsTOF) and ultra large, customized protein databases. However, the proteomic coverage achieved in this study reflects the analytical capacity of the Synapt G1 platform using MSE acquisition by using Uniprot revised databases, providing high confidence identification of the most abundant and representative proteins related to dominant mechanisms inside the oral cavity during the metabolic disorders. The focus of this study is the exploration of trends in host–microbiota crosstalk, rather than an exhaustive mapping of the proteome. The high proportion of differentially abundant proteins (DAPs) among the total identifications suggests that the metabolic disturbances studied (MetS and T2DM) exert a profound impact on the most abundant components of the salivary environment. By utilizing a previously validated workflow [27, 29], we ensured that technical variability was minimized, allowing the observed fluctuations to be attributed to the underlying biological dynamics of the host–microbiota interaction rather than instrumental variability.

While our results are scientifically significant, these findings represent associations within the metabolic spectrum rather than causal relationships, as expected from a cross‐sectional design. The proteomic depth results from the Synapt G1 platform technical characteristics and our randomized equimolar pooling strategy. This approach was chosen to identify associate proteins for each metabolic phenotype by minimizing individual noise, although it limits interindividual correlations. As this study captures a static snapshot of host–microbiota crosstalk, the human and bacterial proteins identified should be interpreted as being associated with the metabolic dysfunction observed in the cohort. Future longitudinal studies using higher sensitivity platforms will be essential to further elucidate the causal roles and therapeutic potential of these candidates in Obesity, MetS, and T2DM.

## 5 Conclusions

Our integrated analysis of the human salivary proteome and bacterial secreted metaproteome in Brazilian individuals with normal weight/control, overweight, obesity, MetS, and T2DM reveals a complex host–microbiota interplay that contributes to oral dysbiosis, chronic inflammation, and metabolic dysfunction. Human proteins MYSM1 and GAD65 were significantly downregulated in obese, MetS, and T2DM groups, showing strong negative correlations with BMI, waist circumference, waist‐to‐hip ratio, and HOMA‐IR. These findings suggest impaired anti‐inflammatory and endocrine functions that exacerbate glucose dysregulation. In contrast, salivary CA6 levels were markedly elevated, positively correlating with systolic blood pressure, glucose, and HOMA‐IR, and indicating a role in fostering an acidic, inflammatory oral environment linked to chronic low‐grade inflammation.

In the bacterial metaproteome, TrxC‐2, UMPK, and RsmH were significantly upregulated in individuals with obesity, MetS, and T2DM, correlating positively with anthropometric and metabolic parameters. These bacterial proteins are associated with oxidative stress response, biofilm formation, and enhanced RNA biosynthesis, reflecting microbial adaptation and increased virulence. Notably, TrxC‐2, UMPK, and RsmH exhibited strong negative correlations with MYSM1 and GAD65, but positive correlations with CA6, suggesting a vicious cycle wherein microbial oxidative stress and virulence amplify host inflammation and metabolic dysfunction, while reduced host protective proteins perpetuate dysbiosis.

Collectively, these findings highlight critical molecular interactions between host and microbial proteins that may contribute to the pathogenesis of obesity, MetS, and T2DM. Our results reinforce the importance of elucidating the complex host–microbiota crosstalk within the oral environment, particularly the molecular and functional pathways through which microbial and host factors influence metabolic dysfunction. Future studies should further investigate the mechanistic basis of these interactions and explore how modulation of the oral microbiota may shape host metabolic and inflammatory responses in metabolic diseases. However, some limitations should be considered. The number of volunteers, the use of pooled samples with nested technical replicates, and the cross‐sectional design, may limit the assessment of single‐patient variability, strict replication independence, and causal relationships. Despite the patterns uncovered by the RF framework provide valuable insights, they must be interpreted as exploratory, and the resulting protein rankings should not be presented as definitive clinical biomarkers. Although data leakage in predictive models was strictly controlled using subpool‐stratified cross‐validation, the integration of all analytical runs introduces a level of statistical dependence inherent to discovery‐phase configurations. Nevertheless, future studies including larger cohorts, individual level analyses, and longitudinal approaches will be important to further validate these findings and to better elucidate the role of oral host–microbiota interactions in metabolic diseases. Analysis on advanced high‐resolution MS platforms, as well as the inclusion of bacterial cell enrichment steps prior to metaproteomic analysis, will allow for a more in‐depth delineation of these processes.

## Funding

This research was funded by FAPERJ.

## Conflicts of Interest

The authors declare no conflicts of interest.

## AI Statement

During the preparation of this work, the authors utilized an artificial intelligence (AI) tool solely to assist with the translation and grammatical refinement of the manuscript into English.

## Supporting information




**Supporting File:** prca70050‐sup‐0001‐SuppMat.zip.

## Data Availability

The raw mass spectrometry data are available from the corresponding author upon reasonable request and with the author's permission.
